# The pan-plastome of *Prunus mume*: insights into *Prunus* diversity, phylogeny, and domestication history

**DOI:** 10.3389/fpls.2024.1404071

**Published:** 2024-06-03

**Authors:** Jie Wang, Junhu Kan, Jie Wang, Xinlin Yan, Yi Li, Thida Soe, Luke R. Tembrock, Guoming Xing, Sen Li, Zhiqiang Wu, Minlong Jia

**Affiliations:** ^1^ College of Horticulture, Shanxi Agricultural University, Jinzhong, China; ^2^ Shenzhen Branch, Guangdong Laboratory for Lingnan Modern Agriculture, Genome Analysis Laboratory of the Ministry of Agriculture, Agricultural Genomics Institute at Shenzhen, Chinese Academy of Agricultural Sciences, Shenzhen, China; ^3^ College of Science, Health, Engineering and Education, Murdoch University, Perth, WA, Australia; ^4^ College of Plant Science and Technology, Huazhong Agricultural University, Wuhan, China; ^5^ Department of Agricultural Biology, Colorado State University, Fort Collins, CO, United States

**Keywords:** pan-plastome, phylogeny, population structure, Prunus, P. mume

## Abstract

**Backgrounds:**

*Prunus mume* in the Rosaceae and commonly referred to as mei or Chinese plum is widely used as a traditional ornamental flowering plant and fruit tree in China. Although some population and genetic analyses have been conducted for this species, no extensive comparisons of genetic variation from plastomes have yet been investigated.

**Methods:**

We *de novo* assembled a total of 322 complete *P. mume* plastomes in this study and did a series of comparative analyses to better resolve pan-plastomic patterns of *P. mume*. To determine the phylogeny and domestication history of this species, we reconstructed the phylogenetic tree of *Prunus* genus, and resolved the population structure of *P. mume*. We also examined the nucleotide variation of *P. mume* to find potential DNA barcodes.

**Results:**

The assembled plastomes exhibited a typical quadripartite structure and ranged from 157,871 bp to 158,213 bp in total size with a GC content ranging from 36.73 to 36.75%. A total of 112 unique genes were identified. Single nucleotide variants (SNVs) were the most common variants found among the plastomes, followed by nucleotide insertions/deletions (InDels), and block substitutions with the intergenic spacer (IGS) regions containing the greatest number of variants. From the pan-plastome data six well-supported genetic clusters were resolved using multiple different population structure analyses. The different cultivars were unevenly distributed among multiple clades. We also reconstructed a phylogeny for multiple species of *Prunus* to better understand genus level diversity and history from which a complex introgressive relationship between mei and other apricots/plums was resolved.

**Conclusion:**

This study constructed the pan-plastome of *P. mume*, which indicated the domestication of *P. mume* involved multiple genetic origins and possible matrilineal introgression from other species. The phylogenetic analysis in *Prunus* and the population structure of *P. mume* provide an important maternal history for *Prunus* and the groundwork for future studies on intergenomic sequence transfers, cytonuclear incompatibility, and conservation genetics.

## Introduction

The Rosaceae family is extensively distributed in temperate regions and comprises *ca.* 120 genera and 3,300 species several of which are of major commercial importance (Zhang et al., 2017). *Prunus* is a large genus in the Rosaceae containing approximately 250–400 species of trees and shrubs that are found mainly in the northern temperate zone, subtropical zones, and tropical regions (Rehder, 1956), with Eastern Asia being an important center of diversity (Chin et al., 2014). Several species of *Prunus* are highly prized for their economic qualities as food crops, such as peach, plum, almond, and sweet cherry (Bortiri et al., 2001). In addition to their value as fruits these species also hold a significant place in gardens all over the world thanks to their beautiful vibrant fruits, leaves, and flowers (Zheng et al., 2022). These species are also considered an important source of timber and medicine (Andro & Riffaud, 1995; Lee and Wen, 2001; Wen et al., 2008). *Prunus* species possess various inflorescence types including racemes, corymbs, and solitary flowers, allowing them to be an ideal system to study the inflorescence evolution (Su et al., 2023). However, the taxonomy of *Prunus* species, particularly proper species delimitation remains unclear (Rehder, 1956; Lee and Wen, 2001; Shi et al., 2013). It has been widely accepted that *Prunus* comprises five subgenera, including *Amygdalus*, *Cerasus*, *Lauro-cerasus*, *Padus*, and *Prunus* (Rehder, 1956; Wen et al., 2008; Chin et al., 2010; Zhao et al., 2016). However, ongoing hybridization between *Prunus* species during evolutionary history has made it extremely difficult to resolve phylogenetic relationships (Chin et al., 2010, 2014). By employing various genomic regions, including plastid markers, nuclear ribosomal ITS, and other nuclear loci, researchers over the last 20 years have attempted to reconstruct the evolutionary relationships of *Prunus* (Bortiri et al., 2001, 2002, 2006; Wen et al., 2008; Chin et al., 2010; Shi et al., 2013; Chin et al., 2014; Zhao et al., 2016; Hodel et al., 2021). In recent years, several nuclear genomes from *Prunus* have been released, including *P. mume* (Zhang et al., 2018), *P. avium* (Wang et al., 2020), *P. dulcis* (Alioto et al., 2020), *P. persica* (Verde et al., 2013), *P. yedoensis* (Baek et al., 2018), *P. domestica* (Callahan et al., 2021), *P. armeniaca*, and *P. salicina* (Jiang et al., 2019; Liu et al., 2020), which laid a foundation to analyze numerous important characters and the phylogeny of *Prunus*.

*Prunus mume* commonly referred to as mei and Chinese plum has been widely used as a traditional ornamental flowering plant and fruit tree in China for thousands of years (Chen, 2010). The earliest cultivation of *P. mume* originated from the Yangtze River Basin, southern China around 4000–5000 years ago and spread throughout East Asia (Zhang, 1987). *Prunus mume* is a woody decorative plant of early spring that has long been regarded as a symbol of Chinese culture (Zhang et al., 2012), which is noted for colorful corollas, diverse flower morphology, and an ability to tolerate low temperatures (Chu, 1999a). *Prunus mume* has been classified into eleven cultivar groups based on morphological differences and include Pendulous, Single Flowered, Versicolor, Pink Double, Flavescens, Tortuosa, Green Calyx, Alboplena, Cinnabar Purple, Apricot Mei and Meiren (Chen, 2010). The history of these cultivars has involved complex gene introgression and multiple hybridization events among different cultivars of *P. mume* (Zhang et al., 2018).

With the continuous update and development of sequencing technology, T2T genomes of many species have also been published, which will promote the continuous in-depth research of genomes (Lan et al., 2023, 2024). Plastome data is regularly used in phylogenomic and population genomic studies now due to the reliability of assembling these genomes and the large comparative databases for analyses (Wang et al., 2023). Most plastomes have a conserved circular structure, comprising two inverted repeats (IRs) separated by a large single copy (LSC) and a small single copy (SSC) region (Jansen & Ruhlman, 2012). They usually comprise 120–130 genes that are linked to photosynthesis, transcription, and translation (Wang et al., 2024). Most plastomes are parthenogenetic, haploid, and non-recombinant, making them applicable in complicated lineages with incomplete lineage sorting and frequent introgression (Biersma et al., 2020; Jiang et al., 2023). Moreover, due to plastomes moderate mutation rate (higher than mitochondrial genes but lower than nuclear genes), they are widely utilized in comparative genomics research and evolutionary studies (Wu & Ge, 2012; Dong et al., 2020; Lin et al., 2023). Compared with traditional single plastome studies, multiple plastome datasets across a species have substantially advanced studies of agronomic plant species. Some recent examples include a study of 321 complete plastomes used to distinguish pepper (*Capsicum*) cultivars (Magdy et al., 2019), 343 complete plastomes within Petato section of *Solanum* which provided new insights into potato diversity, phylogeny, and species differentiation (Yan et al., 2022), as well as studies of pan-plastomes constructed for species such as *Hemerocallis citrina* (Jia et al., 2024). *Nelumbo nucifera* (Wang et al., 2022), *Fagopyrum tataricum* (Zhou et al., 2023), *Oryza* (He et al., 2021), and *Beta vulgaris* (Sielemann et al., 2022). However, no such investigation has been conducted for *P. mume*.

Here, we *de novo* assembled a large plastome dataset consisting of 322 complete *P. mume* plastomes to: (a) construct a reliable pan-plastome for *P. mume*; (b) identify the hypervariable regions of plastomes for developing potential molecular markers; (c) resolved the phylogenetic relationships and population structure of *P. mume* to comprehensively survey the diversity in this species; and (d) reconstructed phylogenetic relationships of *Prunus* to infer the evolutionary history of *Prunus* species especially as relates to introgression with *P. mume*.

## Materials and methods

### Plant materials, plastome assembly, and genome annotation

We *de novo* assembled a total of 322 complete *P. mume* plastomes from the whole-genome resequencing data, which were downloaded from the ENA database (https://www.ebi.ac.uk/ena/browser/; Study accession No. PRJNA352648)(**Supplementary Table S1**) (Zhang et al., 2018). FastQC v0.11.5 (http://www.bioinformatics.babraham.ac.uk/projects/fastqc/) was utilized for data quality control and Trimmomatic (Bolger et al., 2014) was employed to acquire clean reads. The obtained clean raw WGS reads were aligned to a published *P.mume* plastome (GenBank accession: MN101214.1) to filter plastid-origin reads with bwa v0.7.17 (Li & Durbin, 2010) and SAMtools v1.9 (Li et al., 2009). Then, SPAdes v3.15.2 (Bankevich et al., 2012) and Bandage v0.8.1 (Wick et al., 2015) were used for *de novo* assembly of whole plastomes from resulting plastid-origin reads. MAFFT v.7 (Katoh & Standley, 2013) was used to confirm that the assembled plastomes were oriented correctly. The LSC and SSC orientations were manually adjusted to maintain collinearity in the resulting plastome sequences using MEGA7 (Kumar et al., 2016), The total length and GC content of different plastome structures, including LSC, SSC, and IR regions, were calculated using a customized Perl script to provide assembly metrics. Genome annotation of the plastomes was performed using the online program GeSeq (Tillich et al., 2017), and manually reviewed and modified as needed. Finally, the program Chloroplot (Zheng et al., 2020) was used to visualize the pan-plastome map of *P.mume*.

### Haplotype and genetic diversity analyses

Using the corrected alignment of all plastome sequences, the haplotypes of plastomes were ascertained in DnaSP 6 (Rozas et al., 2017), with the option to eliminate gaps and missing loci. Using TCS (Clement et al., 2000) and the median-joining approach, haplotype networks were inferred and displayed in Popart v1.7 (Leigh et al., 2015) to examine the ancestry of the identified haplotypes. The output figure was further modified to be more readable in Adobe Illustrator software (Adobe Systems Incorporated, USA). The programs DnaSP 6 (Rozas et al., 2017) and MEGA7 (Kumar et al., 2016) were used to calculate the haplotype diversity (Hd) and nucleotide diversity (Π) for each genetic cluster of haplotypes, and the evolutionary distances based on the Tamura-Nei distance model was used to calculate population differentiation index (Fst), between different genetic clusters by using the plastomic SNVs. The principal coordinates analysis (PCA) was conducted in TASSEL 5.0 (Bradbury et al., 2007) and the PCA diagrams were drawn in the package ggplot2 in R v 4.2.1 (Ginestet, 2011).

### Phylogenetic and population structure analyses

To resolve the phylogenetic status of *P. mume*, we combined 322 newly assembled *P. mume* plastomes and 151 additional *Prunus* plastomes from several species which were downloaded from NCBI database (https://www.ncbi.nlm.nih.gov/) (**Supplementary Table S2**). To perform the phylogenetic analysis, complete sequences of plastid genomes were aligned in MAFFT 7 (Katoh and Standley, 2013) and manually adjusted in MEGA7 (Kumar et al., 2016). Subsequently, maximum likelihood (ML) trees were constructed by IQtree v.2.1 (Nguyen et al., 2015). The visualization of phylogenetic trees was performed by FigTree v.1.4.2 (http://beast.bio.ed.ac.uk/FigTree). SNV-sites were used to derive a SNV dataset from the entire-plastome alignment (Page et al., 2016). Based on the SNV data matrix and with K values ranging from 1 to 12, population structure analysis was carried out using Structure v.2.3.4, with 10 replicates for each K value (Hubisz et al., 2009). CLUMPP (Jakobsson & Rosenberg, 2007) was utilized to calculate the best K value, and the ggplot2 package of R v 4.2.1 (Ginestet, 2011) was used to display the results.

### Nucleotide variant analysis across the *Prunus mume* pan-plastome

Using the complete plastome alignments, nucleotide variants were identified for *P. mume* plastomes, using the full plastome alignments. Single nucleotide variants (SNVs), block substitutions (two or more consecutive nucleotide variants), nucleotide insertions or deletions (InDels), and mixed sites (which comprise two or more of the preceding three variants at a given site) are the four categories into which nucleotide variants were separated. Graphs produced by variant analyses were created using R v4.2.1’s ggplot2 package.

## Results

### Phylogenetic relationships of *Prunus*

**Figure 1:**
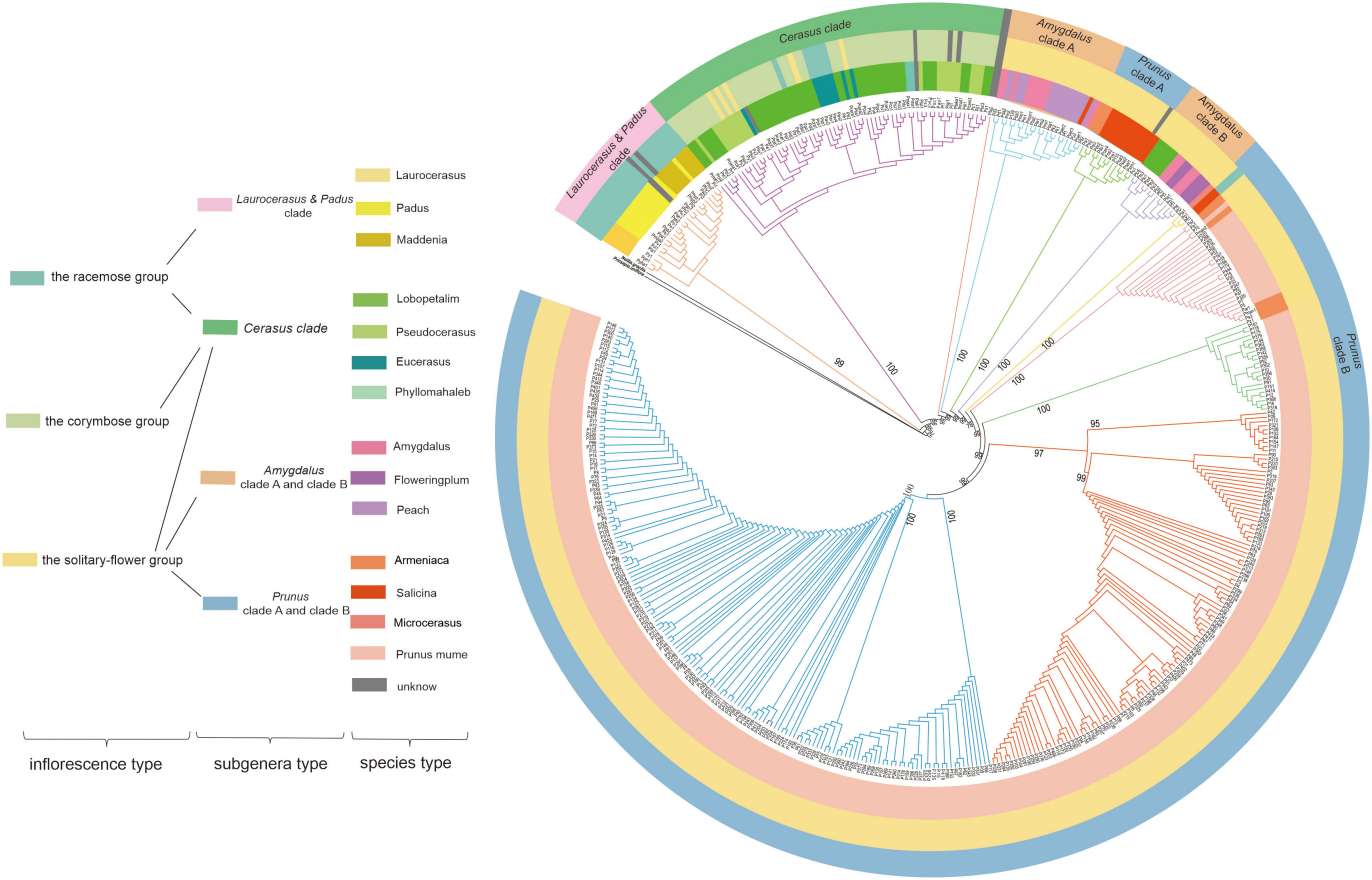
ML tree of section *Prunus* based on whole plastid genome dataset. To the left of the tree is a color-coded legend indicating the different categories into which the samples were placed. The first column refers to the inflorescence type, the second column refers to the subgenera type and the third column refers to the species type.

To reveal the evolutionary relationship among *Prunus*, an ML tree was constructed based on our 322 newly assembled *P.mume* plastomes combined with complete plastomes from an additional 151 *Prunus* species. *Neillia gracilis* and *Prinsepia uniflora* were used as outgroups. As shown in **Figure 1**, *Prunus* was divided into six main clades. According to the previous classification of *Prunus* (Rehder, 1956; Wen et al., 2008; Chin et al., 2010), there were five subgenera in this genus, referred to as *Laurocerasus*, *Padus*, *Cerasus*, *Amygdalus* and *Prunus* (Zhao et al., 2016) (**Figure 1**). Since our phylogenetic tree indicated that *Laurocerasus* and *Padus* are not monophyletic, we further grouped them together into a clade, called the *Laurocerasus* & *Padus* clade. Therefore, six major clades were resolved, corresponding to *Laurocerasus* & *Padus* clade, *Cerasus* clade, *Amygdalus* clade A, *Amygdalus* clade B, *Prunus* clade A and *Prunus* clade B. *Laurocerasus* & *Padus* clade including species from *Laurocerasus*, *Padus* and *Maddenia.* The *Cerasus* clade included *Lobopetalum*, *Pseudocerasus*, *Eucerasus* and *Phyllomahaleb*. The *Amygdalus* clade included *Amygdalus* the flowering plums and peaches. The *Prunus* clade including *Armeniaca*, *Salicina*, *Microcerasus* and *P. mume*. Due to the large sampling of *P. mume* accessions contained in this ML tree, we found that some *P. armeniaca* and *P. mume* clustered together. Moreover, we also observed that a subset of *Lobopetalum* did not cluster with *Cerasus*, but with *Amygdalus*. To better understand the evolutionary relationship in *Prunus*, apart from the molecular data, we classified it based on the type of inflorescence, including racemes, corymbs, and solitary flowers (**Figure 1**). The species in *Laurocerasus* & *Padus* clade only belonged to the racemose group, while the *Cerasus* clade contained three inflorescence types. We also found subg. *Amygdalus* was divided into the solitary-flower group. The results also shown that subg. *Prunus* mostly belonged to the solitary-flower group, with only *P. salicina* divided into the corymbose group.

### Phylogenetic relationships and haplotype analysis of *Prunus mume*

**Figure 2:**
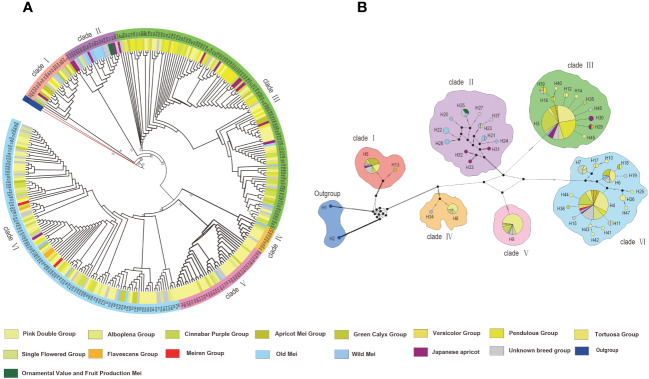
**(A)** ML tree of 322 P*. mume* accessions. **(B)** Haplotype network of *P. mume* accessions. The size of pie chart is proportional to the number of accessions with the same haplotype. The colors in the pie chart indicate the percentage of accessions from different cultivars.

To explore the phylogenetic relationships of 322 different accessions of *P. mume*, we constructed evolutionary trees based on the complete plastomes of all accessions, with *P. padus* and *P. triloba* used as outgroups. 322 P*. mume* accessions were classified into 16 groups, including 11 cultivar groups (Pendulous, Single Flowered, Versicolor, Pink Double, Flavescens, Tortuosa, Green Calyx, Alboplena, Cinnabar Purple, Apricot Mei and Meiren), Old Mei, Wild Mei, Japanese apricot, Ornamental value and fruit production Mei, the accessions that could not be reasonably classified were labelled as unknown breed group (Chu, 1999b; Chen, 2010). Six clades were clearly resolved, which was consistent with the results of haplotype analysis (**Figure 2A**). Except for clade II, which only contains the Single Flowered cultivar group, the other clades contain different types of cultivar groups. Similarly, it cannot be ruled out that there are Single Flowered or other cultivar groups within the unknown breed group included in clade II. As the most abundant variety, the Pink Double group (101) was widely distributed in clades I, III, V, and VI, among which clade VI was the most abundant (38, 37.60%). Alboplena cultivars (11) were also distributed in clades I, III, V, and VI, with the most in clade VI (6, 54.50%). Cinnabar Purple cultivars (45) were distributed in clades I, III, V, and VI, with the most in clade III (25, 55.60%). Apricot Mei cultivars (12) were mainly distributed in clade I, with smaller distributions in clade III and IV. Green Calyx cultivars (13) were distributed in clade III, V, and VI with the same abundance in clade III and clade VI, but only one in clade V. Versicolor cultivars (5) were distributed in clade III and clade VI. Pendulous cultivars (30) were distributed in clades I, III, and VI. The majority of Pendulous cultivars were found in clade III (25, 83.30%), followed by clade VI (5, 16.70%), while clade I contained only one. Tortuosa cultivars were solely distributed within clade VI, which contains only one accession. The Flavescens and Meiren cultivars were also only distributed in clade VI. Single Flowered cultivars (24) were distributed among all clades, with the greatest number in clade VI and only one each in clades I, II, and IV. Old Mei cultivars (7) were distributed in clades I, II, III and VI, with most distributed in clade II (4, 57.14%). Wild Mei cultivars (12) were distributed in clades II, III, IV, V, and VI, although mainly in clade II (5, 41.70%). Ornamental value and fruit production Mei cultivars were only distributed in clade II which contained two accessions. Japanese apricot cultivars (14) were distributed in clades I, II, III, and VI, with most in clade III (8, 57.14%) and the fewest in clade VI (1, 7.14%).

By utilizing an alignment matrix encompassing 322 P*. mume* plastomes, we conducted a SNV-based haplotype analysis, resulting in the identification of 46 haplotypes. The two haplotypes (h1-h2) represented outgroups, which did not belong to *P. mume* and appeared to show a large number of nucleotide variations. The h1 stands for *P. padus*, the h2 stands for *P. triloba*. *Prunus mume* accessions were clearly divided into six genetic clusters, which agreed with the phylogenetic tree (**Figure 2B**). There were two haplotypes in clade I, 12 in clade II, 11 in clade III, two in clade IV, one in clade V, and 17 in clade VI. The two haplotypes (h3-h4) may be old haplotypes.

### Population structure and genetic diversity of *P. mume*

**Figure 3:**
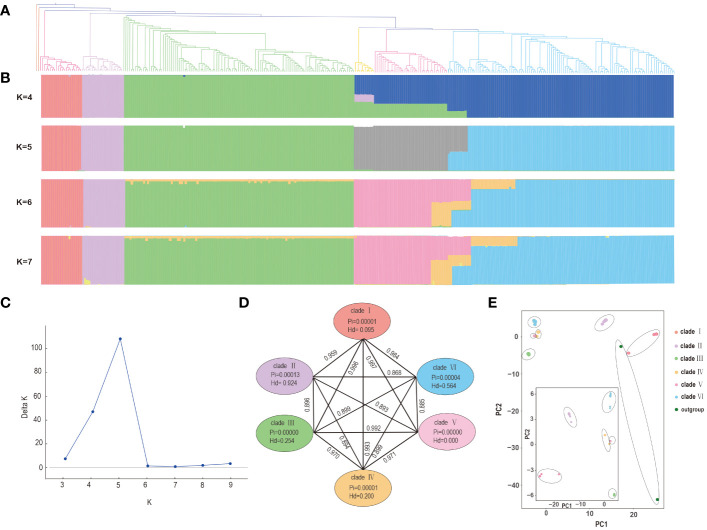
Phylogenetic and population structure analyses of *P. mume*. **(A)** The structure of phylogenetic topology in Bayesian inference. **(B)** a bar-plot of all samples showing the percentage assignment to a given group across different values of K **(C)** Delta K across a range of K values from 3−9. **(D)** Genetic diversity and differentiation of six clades of *P. mume*. Pairwise F_st_ between the corresponding genetic clusters is represented by the numbers above the lines joining two bubbles. **(E)** PCA analysis including outgroup (larger panel) and excluding outgroup (inset panel) showing the first two components in both cases.

To gain a deeper understanding of the matrilineal history of *P. mume*, population structure was inferred by STRUCTURE with K = 5 based on an SNV-only input matrix being the best supported partition (**Figure 3C**). Our results showed that different K values indicate difference in group stability of *P. mume* lineages. At all K values the first three clades were resolved in the bar plot in all cases. Similarly, mixed membership was observed at all K values in some of the clades beyond the earliest diverging three. (**Figures 3A–C**). Previous studies have shown introgression between *P. mume* cultivars and other *Prunus* species [17]. Shared membership in the STRUCTURE barplots using plastome data also suggest that introgression may have occurred during *P. mume* domestication but because plastomes are uniparentally inherited this might also be the result of incomplete lineage sorting. (**Figures 3A, B**). Principal component analysis (PCA) was conducted to further investigate the genetic diversity based on all 322 accessions with the SNV dataset (**Figure 3E**). The results of PCA using whole plastome alignments were similar to those seen in other population analyses conducted herein. Nucleotide and haplotype diversity among the six clades of *P. mume* showed minor variations. Nucleotide diversity (Pi) differed among different clades (**Figure 3D**). The highest nucleotide diversity (Pi) was in clade II (Pi=0.00013), followed by clade VI (Pi=0.00004), clade IV (Pi=0.00001) clade I (Pi=0.00001), and clade III (Pi=0.00000) and clade V (Pi=0.00000). The highest haplotype diversity (Hd) was in clade II (Hd = 0.924), followed by clade VI (Hd = 0.564) and clade III (Hd = 0.254), clade IV (Hd=0.200), and clade V (Hd=0.000). Our analyses of genetic divergence between 322 P*. mume* accessions were found in F_st_ values. Among all clades, clade III had the highest level of divergence (III to I F_st_ = 0.996; III to II F_st_ = 0.896; III to IV F_st_ = 0.970; III to V F_st_ = 0.992; and III to VI F_st_ = 0.899). Clade II also exhibited high levels of divergence. Clade III and clade V showed lower levels of divergence compared to the other clades.

### Plastome structure and organization of *Prunus mume*

**Figure 4:**
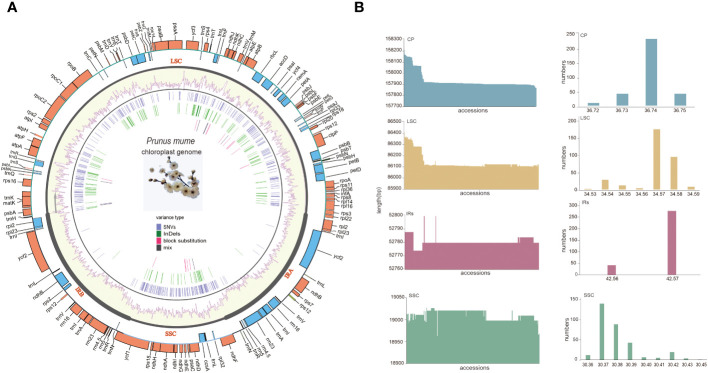
The pan-plastome of *P. mume*. **(A)** The general plastome structure *P. mume*. The outer circle shows gene placement and annotation across the genome, the transcribed genes that run clockwise and counterclockwise are indicated by the light blue and orange bars respectively. The GC content is displayed as yellow bars inside of the tetrad divisions (LSC, SSC, IRA, and IRB) in the second outer circle. SNVs, InDels, block substitutions and mixed variants are represented within purple, green, red and black lines, respectively. Single nucleotide variants (SNVs), block substitutions (BS, two or more consecutive nucleotide variants), nucleotide insertions or deletions (InDels), and mixed sites (which comprise two or more of the preceding three variants at a particular site) are the four categories into which variants are divided. **(B)** Sequence length and the GC content barplots of the total plastome, LSC, SSC and IRs.

All *P. mume* plastomes exhibited a typical quadripartite and circular organization. The size of the complete plastomes ranged from 157,871 bp to 158,213 bp (Med = 157,910 bp). Among the different plastome regions, the LSC size ranged from 86,102 bp to 86,412 bp (Med = 86,115 bp), the SSC size ranged from 18,963 bp to 19,011bp (Med = 19,011 bp) and the IR size ranged from 26,387 bp to 26,400 bp (Med = 26,390 bp). The total GC content (%) of the complete plastomes ranged from 36.73 to 36.75 (Med = 37.74), with 34.53 to 34.59 (Median = 34.57) for the LSC, 30.36 to 30.45 (Median = 30.38) for the SSC, and 42.56 to 42.57 (Median = 42.56) for the IRs. The GC content of the IR regions was higher than that of the LSC and SSC (**Supplementary Table S3**, **Figure 4**).

A total of 112 unique genes were annotated and grouped into functional categories as follows: 78 protein-coding genes (PCGs), 30 transfer RNA (tRNA) genes, and 4 ribosomal RNA (rRNA) genes (**Figure 4A**). The distribution of these identified genes in the LSC, SSC, and IRs was as follows: a total of 83 genes in the LSC, including 62 PCGs and 21 tRNA genes; 13 in the SSC, including 12 PCGs and 1 tRNA gene, and 16 duplicated in the IRs, including 5 PCGs, 7 tRNA genes, and 4 rRNA genes (all genes in the IRs were counted once). Among these genes 18 (13 located in LSC,1 in SSC and 4 in the IRs) including 12 PCGs (*atpF*, *clpP*, *ndhA*, *ndhB*, *petB*, *petD*, *rpl2*, *rpl16*, *rpoC1*, *rps12*, *rps16*, and *ycf3*) and 6 tRNA (*trnA-UGC*, *trnG-UCC*, *trnI-GAU*, *trnK-UUU*, *trnL-UAA*, and *trnV-UAC*) contained introns. In particular, *clpP*, *ycf3*, and trans-spliced *rps12* (characterized by the first exon locating in LSC and the other two in the IRs) contained two introns (**Supplementary Table S4**, **Figure 4B**).

### Analysis of nucleotide variation in *Prunus mume* plastomes

**Figure 5:**
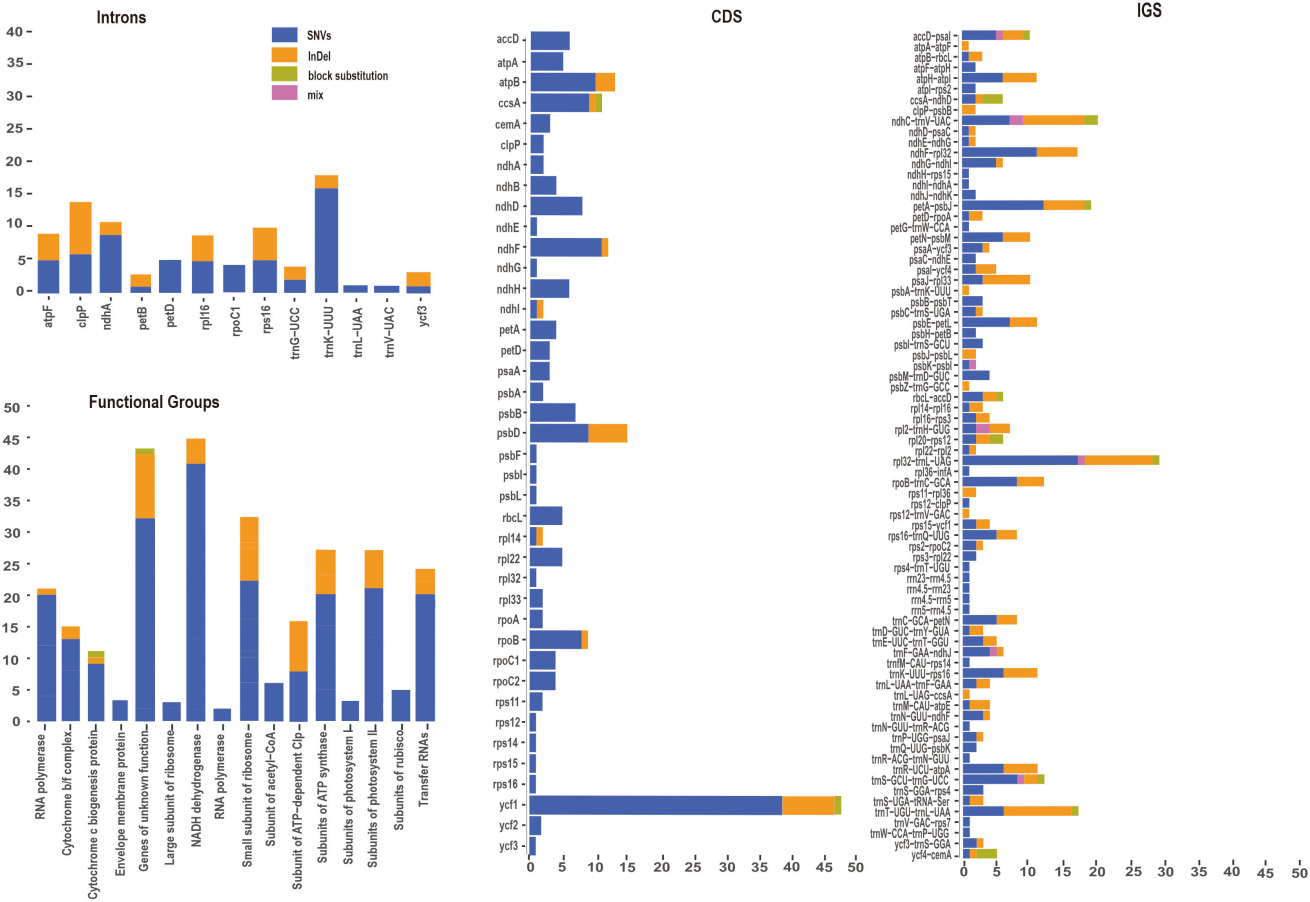
Variant locations categorized by genic position (CDS, Introns and IGS) and functional group.

To gain a deeper understanding of nucleotide variation of the *P. mume* pan-plastome, 322 newly assembled plastomes were aligned for following analyses. A total of 682 variants were identified among coding sequences (CDS) and intergenic spacers (IGS) of 322 plastomes. Variants were classified as SNVs, InDels, block substitutions and mixed variants. Among these variants, SNVs had the largest number and proportion (455, 66.72%), followed by InDels (201, 29.50%), block substitutions (18, 2.64%) and mixed variants (8, 1.17%) (**Figure 5**, **Supplementary Table S5**). Variants were dispersed unevenly across the pan-plastome. The LSC (474, 69.50%) included the greatest number of variants, followed by the SSC (161, 23.61%), and IRs (47, 6.90%) contained the fewest variants. When analyzing the variants by genic position, the IGS regions had the highest number of variants (381, 55.87%), followed by CDS (220, 32.26%).

Among the CDS, *ycf1* (48 total variants) had the most variants, including SNVs (38), InDels (7) and block substitutions (1). *ccsA* (11) also had three types of variations, including SNVs (9), InDels (1) and block substitutions (1). Eleven genes including *ycf3*, *rps12*, *rps14*, *rps15*, *rps16*, *rpl32*, *psbF*, *psbL*, *psbI*, *ndhG*, and *ndhE* had only one SNV each (**Figure 5**). Seven genes including *psbA*, *rpl33*, *clpP*, *rpoA*, *rps11*, *ndhB* and *ndhA* had two SNVs, *rpl14* and *ndhl* each included one SNV and one InDel The genes *rpoB* included eight SNVs and one InDels, *psbD* included nine SNVs and six InDels.

### Hypervariable regions across the *Prunus mume* pan-plastome

**Figure 6:**
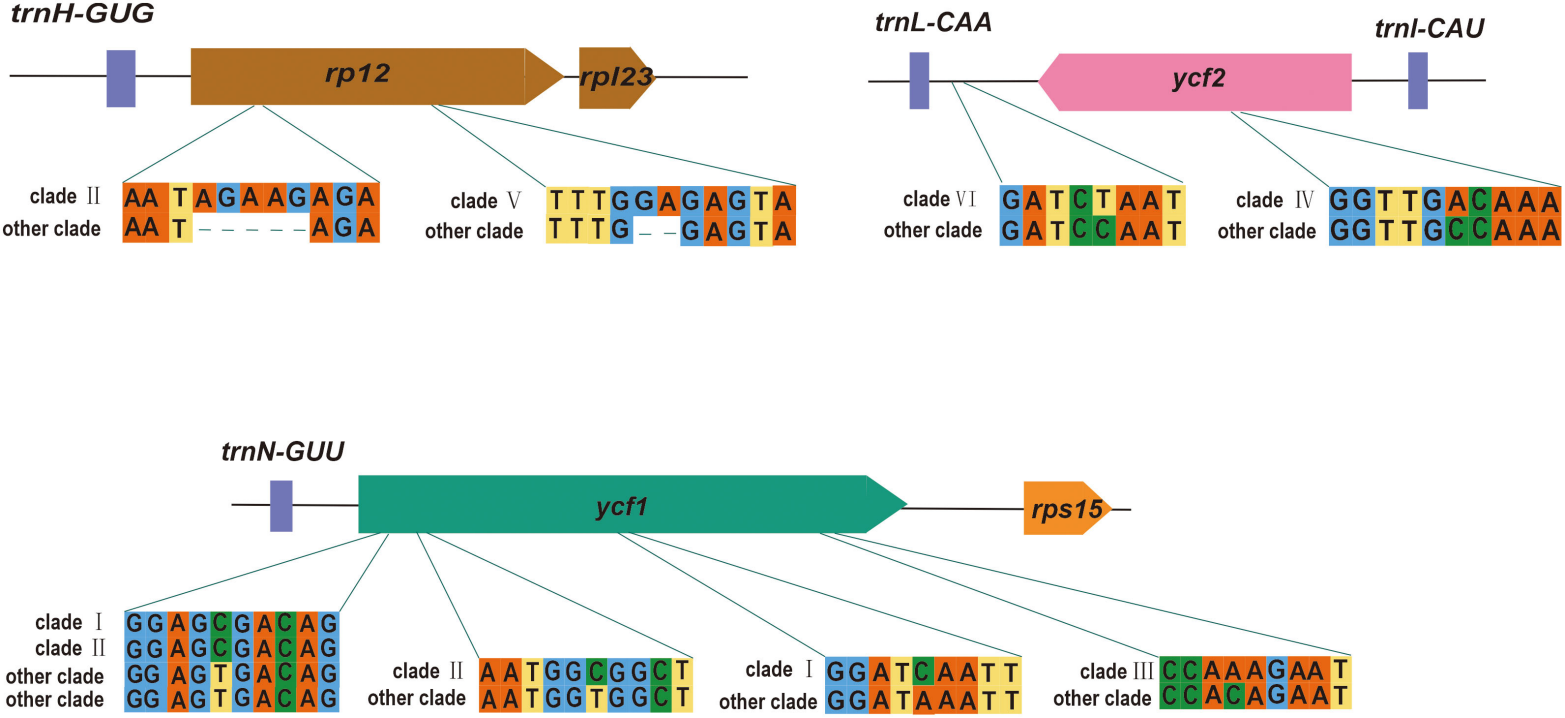
Examples of variable sites.

Numerous nucleotide mutations were observed throughout the *P. mume* plastome, despite structural and gene conservation. These nucleotide variations have the potential to be employed in the development of molecular markers. According to the evolutionary tree, we found several potential loci to distinguish each clade (**Supplementary Table S4**). Clade 1 contained the most hypervariable regions, followed by clade II. Here, we selected genes located in the boundary region to show the sequence differences for each clade (**Figure 6**). We found that *ycf1* had a large number of variant loci, which contained unique SNVs to clade I, II and III. There were also some SNVs that clade I and II exclusively contained. We also found some loci that contain unique InDels, such as *rpl2*, which contained the unique InDels to clade II and clade V. We also found loci which have unique SNVs in the IGS, such as *trnL-CAA-ycf2* containing unique SNVs to clade VI. The high variability in IGSs could improve resolution of intraspecific studies.

## Discussion

### Phylogenetic relationships in *Prunus*

Our research combined plastome data of *Prunus* to resolve evolutionary relationships in the genus more broadly to better understand the evolution of inflorescences and possible past maternal introgression. Previous studies have indicated that the racemose, solitary-flowering, and corymbose inflorescences may provide signal for delimiting *Prunus* lineages. Past plastomic studies, have determined that the *Prunus* species in corymbose group had a close relationship with the solitary-flower group and the corymbose group was resolved as sister to the racemose group, which was the ancestral state of inflorescence in *Prunus* (Chin et al., 2010, 2014; Zhao et al., 2016). Our reconstructed phylogenetic tree in *Prunus* also exhibited a similar pattern as these results although some polyphyly in the inflorescence type mapped onto our plastome tree suggests a complex history and evolution among *Prunus* species. We also suggest that *Padus* and *Lauroploidus* may have originated from allopolyploidy and hybridization events for both are not monophyletic and clustered into a clade. Moreover, our study also provided strong evidence for supporting the placement of *P. maackii* in subg. *Padus* (Zhao et al., 2016; Su et al., 2023). Some studies have suggested that two species from *Microcerasus* should be divided into the solitary-flower group instead of the corymbose group (Bortiri et al., 2001; Lee and Wen, 2001; Chin et al., 2014). Genetic analyses based on different types of molecular data also supported this opinion (Bortiri et al., 2002, 2006; Liu et al., 2012; Shi et al., 2013; Zhao et al., 2016). Our reconstructed phylogenetic tree was congruent with these previous studies and also did not support the inclusion of *Microcerasus* into the Cerasus group (Kataoka et al., 1988). All species in the corymbose group were from the subgenus *Cerasus*. The *Microcerasus* species were recorded scattering in the solitary flower group (Shi et al., 2013), and our results also support this conclusion. Furthermore, it was noted that *Microcerasus* species bred with solitary-flower group species more frequently than with *Cerasus* group species (Mowrey and Werner, 1990). According to morphological and genetic data, the classification of *Microcerasus* into the solitary-flower group was reasonable (Baack and Rieseberg, 2007). To further understand the origin (s) and phylogenetic status of *Microcerasus* species, future research should sample these species as well as closely related taxa more intensively. The phylogenetic relationships among *P. mume*, *P. armeniaca*, and *P. salicina* are still a matter of debate, due to frequent hybridization, apomixis and a complex history of cultivation (Baack and Rieseberg, 2007; Xue et al., 2019; Numaguchi et al., 2020). We found that some apricots mei and *P. salicina* clustered together, suggesting that past introgression was still retained in *P. armeniaca* and *P. mume*. Based on the maternal inheritance of plastome and biparental inheritance of nuclear genome, apricots, Mei, and *P. salicina* may be originated from the same paternal parent and several maternal parents through past introgression.

### Hypervariable regions in the *P. mume* pan-plastome

In this study, we employed 322 newly assembled plastomes to construct the pan-plastome of *P. mume*. Based on these plastome data, we conducted comprehensive analyses, such as structural feature and nucleotide variation. Similar to other research, our study revealed a significant conservation of quadripartite structure, sequence length, gene order and GC content in *P. mume* plastomes (Daniell et al., 2016). The IR regions exhibited higher GC contents than the LSC and SSC regions, which could contribute to their greater conservation (Khakhlova and Bock, 2006; Fan et al., 2018). Despite the conserved characteristics of plastome sequences, there are still abundant polymorphisms among plastomes for effective phylogenetic analysis and species identification (Gitzendanner et al., 2018; Xue et al., 2019). By comparing the plastomes in a specific genus or species, one can identify potential DNA barcodes for genus/species identification. These DNA barcodes often exhibit higher accuracy and discriminatory power in plant identification compared to universal barcodes (Dong et al., 2021). In the nucleotide variation analysis of plastomes of *P. mume*, *ycf1* had the highest number of variants in the CDS region. Additionally, *psbD* and *atpB* were found to have a greater number of variants than other genes and they could be employed as potential DNA barcodes to distinguish different accessions of *P. mume.* The IGS regions were determined to be more variable than the CDS regions in plastomes from other studies (Song et al., 2020). Likewise, InDels are more common beyond CDS regions, and the *P. mume* pan-plastome also exhibited a similar pattern of variant abundance and type as other plastomes (Wang et al., 2022). As more research on pan-plastomes is completed, intronic regions and hypervariable regions from IGSs are also regarded as valuable resources for species identification at the intraspecific level, with higher resolution compared to some common DNA barcode from CDS regions, such as *rbcL* and *matK*. This study proved that some IGS regions: *rpl32-trnL-UAG* and *ndhC-trnV-UAC* were especially rich in informative markers. Similar to pan-plastomes of *Brassica napus*, *Nelumbo nucifera*, our pan-plastome research revealed that SNVs are by far the most prevalent form of mutation (Song et al., 2020; Tao et al., 2021; Wang et al., 2022). Nowadays, the plastomes’ crucial role in evolution is more deeply understood by researchers and further pan-plastome research from a variety of cultivated taxa will no doubt clarify the history of domestication in *P. mume*. Further pan-plastome research from a variety of cultivated and wild taxa will allow for improved descriptions of patterns unique to domesticated lineages. Such work will improve the development of elite varieties.

### Phylogenetic relationships and divergence among clades of *Prunus mume*

Numerous processes, such as genetic drift, reproductive isolation, local adaptation, demographic fluctuations, mode of reproduction, artificial selection, and human domestication, are known to have an impact on plant population structure and genetic diversity (Lovette, 2004). It is commonly believed that plastomes lack any genes related to domestication. The nucleotide diversity of plastomes is expected to be reduced due to severe artificial selection during domestication (Haudry et al., 2007; Gross and Olsen, 2010; Avni et al., 2017; He et al., 2021) however this may not be the case in long-lived woody species. According to early records, Chinese plum landraces were selected from a wild lineage of *P. mume*, the true mume branch. In addition to the true mume lineage, two hybrid lineages are also known between species *P. mume* and *P. armeniaca* as well as *P. mume* and *P. cerasifera* (Zhang et al., 2018). From our phylogeny the nearly all *P. mume* samples appeared to be from *P. mume* maternal origins although a few samples within the *P. mume* lineage were identified as *P. armeniaca* suggesting past introgression. Such imbalance in *P. mume* plastomes despite known instances of interspecific introgression may suggest that unidirectional nucleocytoplasmic incompatibly (Guo et al., 2019; Wang et al., 2021) has occurred in the hybridization history of *P. mume* but more sampling needs to be done before this can be concluded with confidence. If such a pattern of nucelocytoplasmic incompatibly occurred in *P. mume* domestication this will be valuable to know in future breeding projects to avoid mismatching parents.

From our reconstructed phylogenetic tree based on pan-plastomes of *P. mume*, six well-supported clades were identified and each clade differed in terms of haplotypic diversity and the cultivar group membership (**Figure 2**). The morphological characteristics demonstrated significant variations among different cultivar groups within *P. mume*. We also found multiple cultivar groups instead of any single one distributed in the same clade. Although, most wild Mei and old Mei were distributed in clade II, and a majority of cultivar accessions of *P. mume* were distributed in clades I, III, IV, V and VI. There were a large number of haplotypes located in clade II and most haplotypes contained only one accession. We speculate that clade II was a wild lineage due to this disruption pattern. These discoveries could help clarify the patterns of introgression during *P. mume* domestication. These findings also support the reliability of whole plastome data in studying plant diversity at multiple taxonomic levels, especially at the intraspecific level. In summary, these pan-plastome resources will not only provide new insights into the natural and domestication history of *Prunus*, but also advance the progress of research on organelles.

## Conclusions

Our understanding of the evolution and phylogeny of section *Prunus* have been greatly aided by numerous studies (Zhao et al., 2016; Xue et al., 2019; Ding et al., 2022; Su et al., 2023). However, a lack of pan-plastomic studies have hindered our understanding of matrilineal relationships within this important genus of plants. The current study where we have *de novo* assembled 322 different *P. mume* plastomes and 151 other *Prunus* accessions plastomes to reconstruct the *Prunus* evolutionary tree is an important contribution in understanding natural plastome evolution as well as plastome evolution under artificial selection within the cultivated *P. mume* lineage. For instance, we found that six well-supported plastome lineages could be resolved within *P. mume* and that most named cultivar groups were selected from two of these clades. Furthermore, we resolved polyphyly between *P. mume* and *P. armeniaca* indicating past introgression in the domestication of these apricots. Lastly our comprehensive description of nucleotide variants in the mei pan-plastome will aid future researchers and breeders in understanding the molecular evolution of the plastome in this important fruit species and utilize this knowledge in trait improvement and preservation of wild and cultivated germplasm.

## Data availability statement

The original contributions presented in the study are included in the article/**Supplementary Material**. Further inquiries can be directed to the corresponding authors.

## Author contributions

JW(1^st^ author): Writing – original draft. JK: Writing – review & editing, Methodology. JW(3^rd^ author): Writing – review & editing, Methodology. XY: Data curation, Writing – review & editing. YL: Writing – review & editing, Formal analysis. TS: Data curation, Writing – review & editing. LT: Writing – review & editing. GX: Writing – review & editing. ZW: Writing – review & editing, Funding acquisition. SL: Writing – review & editing, Methodology. MJ: Writing – review & editing, Methodology.
